# How to Get the Most from Methotrexate (MTX) Treatment for Your Rheumatoid Arthritis Patient?—MTX in the Treat-to-Target Strategy

**DOI:** 10.3390/jcm8040515

**Published:** 2019-04-15

**Authors:** Peter. C. Taylor, Alejandro Balsa Criado, Anne-Barbara Mongey, Jerome Avouac, Hubert Marotte, Rudiger B. Mueller

**Affiliations:** 1 Botnar Research Centre, NDORMS, University of Oxford, Windmill Road, Oxford OX3 7LD, UK; 2 Rheumatology unit, University Hospital La Paz, Institute for Health Research–IdiPAZ, Universidad Autonoma de Madrid, 28046 Madrid, Spain; alejandro.balsa@salud.madrid.org; 3 St. Vincent’s University Hospital, Elm Park, Dublin 4, Ireland; anne.b.mongey@ucd.ie; 4 Paris Descartes University, Sorbonne Paris Cité, Rheumatology Department, Cochin Hopital, Assistance Publique-Hôpitaux de Paris, 75014 Paris, France; javouac@me.com; 5 SAINBIOSE, INSERM U1059, Université de Lyon, Saint-Etienne, France Service de Rhumatologie, CHU Saint-Etienne, 42055 Saint-Etienne, France; Hubert.marotte@chu-st-etienne.fr; 6 Division of Rheumatology, Medical University Department, Kantonsspital Aarau, 5001 Aarau, Switzerland; ruediger.mueler@ksa.ch

**Keywords:** methotrexate, rheumatoid arthritis, tolerability, efficacy, posology, titration, oral route, subcutaneous route, bioavailability, effectiveness

## Abstract

Methotrexate (MTX) is a remarkable drug with a key role in the management of rheumatoid arthritis (RA) at every stage of its evolution. Its attributes include good overall efficacy for signs and symptoms, inhibition of structural damage and preservation of function with acceptable and manageable safety, a large dose-titratable range, options for either an oral or parenteral route of administration, and currently unrivalled cost-effectiveness. It has a place as a monotherapy and also as an anchor drug that can be safely used in combination with other conventional synthetic disease-modifying antirheumatic drugs (csDMARDs) or used concomitantly with biological DMARDs or targeted synthetic DMARDs. MTX is not without potential issues regarding toxicity, notably hepatotoxicity and bone marrow toxicity, as well as tolerability problems for some, but not all, patients. But many of these issues can be mitigated or managed. In the face of a welcome expansion in available targeted therapies for the treatment of RA, MTX looks set to remain at the foundation of pharmacotherapy for the majority of people living with RA and other inflammatory rheumatic diseases. In this article, we provide an evidence-based discussion as to how to achieve the best outcomes with this versatile drug in the context of a treat-to-target strategy for the management of RA.

## 1. Introduction

Rheumatoid arthritis (RA) is a chronic autoimmune disease characterized by inflammation, pain, stiffness, and progressive joint destruction with a detrimental impact on joint function, work ability, and health-related quality of life (HrQoL) [1]. The primary goal of treating patients with RA is to maximize long-term HrQoL through control of symptoms, prevention of structural damage, normalization of function, and participation in social and work-related activities. Abrogation of inflammation is considered the most important way to achieve optimal outcomes [2], and its achievement is facilitated by Treat-to-Target (T2T) recommendations [2].

The T2T paradigm has emerged during the last decade and was incorporated in recommendations issued primarily in 2010 and recently updated [2]. The T2T paradigm relies on five principles: the definition of a treatment target; the close and regular assessment of disease activity using composite measures that include joint counts; the regular adaptation of therapy, if the target is not achieved within a particular timeframe; the consideration of individual patients’ aspects; and shared decision-making with the patient. A T2T strategy is more effective in terms of reducing disease activity compared to routine care [3], and has a beneficial effect on working ability. T2T principles underpin the current recommendations of European League Against Rheumatism (EULAR) Task Force [4], American College of Rheumatology (ACR) guidelines [5], and the Canadian Rheumatology Association (CRA) recommendations [6] and the Asian APLAR guidelines [7]. T2T overarching principles affirm the necessity of a shared decision between patient and the whole team involved in the care of RA [2]. 

The ideal treatment target is remission [8], or, if remission cannot be achieved, low disease activity (LDA). Remission is associated with the best achievable outcomes including limitation or prevention of joint damage [9], preservation of physical function, and work capacity as well as optimizing overall quality of life [10,11]. Furthermore, remission reduces comorbidity risks [12]. Treatment targets should however be adapted according to the presence of comorbidities, individual patient factors and drug-associated risks [2] to ensure the most favorable benefit: risk ratio for any given patient. Treatment should be titrated to the therapeutic response assessed by composite measures of disease activity, ideally with therapy adjustment at least every three months [2] if the patient is not in remission, a recommendation based on evidence provided by clinical trials [13,14,15], until the desired treatment target is reached.

Early treatment [16] and rapid attainment of the targeted endpoint are critical. Disease-modifying anti-rheumatic drugs (DMARDs) are the main therapy of RA. The most commonly prescribed conventional synthetic (cs) DMARDs are methotrexate (MTX), leflunomide, sulfasalazine, and hydroxychloroquine [6]. Based on its efficacy, safety, large dose-titratable range, options for either an oral or parenteral route of administration, and cost-effectiveness, MTX holds a unique place in the management of RA, with a role at every stage of the evolution of this chronic condition. MTX monotherapy is recommended as an initial pharmacological strategy [4,5,6], but it can also be used as an “anchor drug” in combination with another conventional synthetic disease-modifying antirheumatic drugs (csDMARD), any biological DMARD (bDMARD), or targeted synthetic DMARD (tsDMARD) [4]. MTX is currently the most commonly used first-line therapy for RA in the world [17].

While recommendations advocate the use of methotrexate at various positions in the contemporary treatment paradigm, the heterogeneity of presentation of RA and versatility of approaches to effective MTX use are such that there is need for evidence- and experience-based supplementary information to enable clinicians to get the most out of MTX for their patients in the T2T era. Notably, this process includes allaying any misconceptions that patients may have concerning this therapy, adapting dose and administration routes for optimal efficacy, mitigating toxicity and tolerability issues, and taking into account the relatively slow kinetic of response, which can be managed by the use of bridging steroids as appropriate.

In this position paper, we address these and other issues regarding the optimal use of MTX in a target population comprising adult (age ≥ 18 years) patients meeting current classification criteria for RA and patients with early inflammatory arthritis suspected of having RA but not yet fulfilling classification criteria. 

## 2. Pharmacology

### 2.1. Pharmacokinetics

MTX (4-amino-10-methylfolic acid), an antifolate agent that was licensed for a RA indication in the 1980s [18], is a prodrug that becomes active when glutamated within cells, exhibiting a high binding activity for dihydrofolate reductase (DHFR) [19].

Oral MTX is absorbed from the small gut by an active transport mechanism involving the proton-coupled folate transporter. Bioavailability ranges from 30% to 70% [20] and plateaus for single oral dose >15 mg [21], suggesting an absorption limitation [22]. Intracellular MTX is gradually polyglutamated (MTXGlu_n_). There is a competitive process between glutamation and deglutamation and steady-state levels of intracellular MTX are reached in a median time of 28 weeks with variability between patients due to different polyglutamation rates [23,24]. The major determinants of MTXGlu_n_ concentrations are age, renal function, and MTX dose [25]. Serum concentration of MTX falls rapidly following an intravenous administration [26]. The plasma half-life is 4.5 h to 10 h [27] but MTX is retained within cells long after its serum clearance. Excretion of MTX is mainly renal, occurring through glomerular filtration and active tubular excretion. Seventy-five percent of MTX is excreted unchanged in the urine with large interpatient variability [28].

The active transport mechanism limits oral MTX absorption resulting in lower bioavailability of higher MTX doses. Interestingly, bioavailability may increase by splitting the oral dose [22] or switching to a parenteral route [22]. Polyglutamation is progressive, explaining the slow onset of the action of MTX [19] and the delay before maximal benefit. In some patients with low glutamation rates, a relatively low dosing strategy will lengthen the time required for adequate drug concentration to be achieved [23]. This may be detrimental, as higher intracellular MTXGlu_n_ levels have been associated with better clinical response [29]. The subcutaneous (SC) administration route is associated with a significant increase in long-chain MTXGlu_n_ when compared to the oral route [30]. Renal function impacts the generation of MTXGlu_n_. The anti-inflammatory effects of MTX in patients with RA are much more prolonged than its plasma half-life might suggests due to the accumulation of polyglutamated metabolites in tissues. Since the MTX excretion mainly involves the kidney, renal failure, or competitive excretion with other drugs in patients receiving multiple treatments will prolong MTX serum half-life [31]. 

### 2.2. Mode of Action

The mechanisms by which low-dose MTX exerts its therapeutic effect in RA remain incompletely understood [19]. MTX has several important anti-inflammatory actions mediated through a variety of pathways that do not involve folate antagonism. The main putative mechanisms are the promotion of adenosine release, inhibition of purine and pyrimidine synthesis, inhibition of methyl donors, generation of reactive oxygen species, downregulation of adhesion-molecule expression, modification of cytokine profiles, and downregulation of eicosanoids and matrix metalloproteinases [19]. Adenosine exerts an anti-inflammatory activity by down-regulating the activation and proliferation of T-Lymphocytes. Adenosine receptors are overexpressed on immune cells and synoviocytes of patients with RA, probably because of the high tumor necrosis factor (TNF) levels. Inhibition of DHFR prevents the regeneration from dihydrofolate to tetrahydrofolate, an essential factor for the production of folate cofactors required for purine and pyrimidine synthesis [32]. Inhibition of DHFR also inhibits the production of methyl donors and attenuates the formation of polyamines. 

Since the anti-inflammatory properties of MTX are not mediated by folate antagonism, some toxic effects may be prevented or minimized by folic acid supplementation (not taken on the same day as MTX) without loss of therapeutic benefit.

In the future, pharmacogenetic advances may help to predict non-responses to MTX, thus directing patients to combination therapy as initial treatment. Promising results have recently been published [33,34].

## 3. MTX: A Forefront Position among csDMARDs

### 3.1. Flexible

More intensive treatment interventions provide the best chance of achieving remission in RA patients [35]. However, potential benefits need to be balanced against potential risks and every effort must be made to ensure acceptable tolerability of any given therapeutic regime. The most frequent strategy for management of early RA is a “step-up” approach, where MTX is initiated at a dose estimated to be well tolerated and then titrated up according to therapeutic response. Current recommendations for management of RA [5,36] advocate the initial use of MTX as a monotherapy with a bridging steroid to give more rapid symptomatic relief before the clinic benefits of MTX become apparent. Some authors have advocated a step-down approach to treatment, initiating MTX at a high dose and arguing that this takes advantage of a “window of opportunity” for best outcomes early in the disease [37]. However, this process has the potential disadvantage of making tolerability issues more challenging to manage. Whatever the strategy, MTX has a central role to play, both as a monotherapy and as an “anchor drug”, for many successful DMARD combinations whether conventional, targeted synthetic, or biologic [38]. Some unique attributes of MTX contributing to this versatility include the wide dose titratable range, choice of administration routes, the favorable efficacy:toxicity ratio, utility in DMARD combinations, potential ancillary benefits, and outstanding cost-effectiveness. Unfortunately, despite treatment guidelines, up to 50% of patients [39] do not continue MTX when treatment with a biologic is initiated. Over the last few years there has been a discussion about tsDMARD or bDMARD monotherapy. Some authors suggest that MTX administered in a tolerated dose should be continued as a combination partner until remission is achieved [40]. This process is recommended because the highest benefits for all targeted therapies are generally observed when they are used with concomitant MTX.

MTX is marketed in the form of tablets with increments of 2.5, 5, or 10 mg/tablet, and as prefilled syringes or auto-injectors with various doses per injection, allowing one to finely tune the weekly dose. Oral and (SC) are the most widely routes used in RA patients. SC injections should be considered for weekly doses of 15 mg or higher due to the better bioavailability of MTX when administered parenterally.

### 3.2. Clinically Efficient

The TEAR study has demonstrated that low disease activity illustrated with the Disease Activity Score-28 with the erythrocyte sedimentation rate (DAS28-ESR) can be achieved by 28% of patients with early RA treated with MTX monotherapy [41]. It was a 2 × 2 factorial design comparing four treatment arms (immediate combination of MTX + etanercept (ETN); immediate combination of MTX + hydroxychloquine (HCQ) + sulfasalazine (SSZ)); initial MTX with step-up treatment adding ETN; initial MTX with step-up treatment adding HCQ and SSZ. The study aimed to assess if it was better to intensively treat all early RA cases with drug combinations or to reserve this procedure for those who do not achieve low disease activity (DAS28-ESR < 3.2) after 24 weeks. At week 24, 28% of the patients from step-up groups achieved the treatment target. Similar results were achieved in the BeST study [42]. An even higher rate was demonstrated by Mueller et al in real-life [43]. Moreover, no difference was found between groups with respect to disease activity after 48 weeks and 102 weeks of treatment. Favorable results obtained with initial MTX monotherapy in the step-up groups were maintained until week 102. Moreover, there was no difference in radiologic progression when comparing step-up with combination therapy in a post-hoc analysis [38]. Since differences in outcomes were negligible between patient groups that were started at a baseline on an aggressive combination therapy regimen, or were delayed before stepping up (by 6 months), the authors concluded that there is no penalty for delaying step-up therapy until it is clinically indicated [38]. In comparison with those patients exhibiting a sustained, good response to MTX monotherapy, aggressive combined treatment would only result in a higher risk of side effects and higher costs. Importantly, these results were obtained employing a strict MTX dosage escalation regime (10 mg/w, escalated to 15 mg/w at week 6 and 20 mg/w at week 12, unless the patients had zero tender joints and zero swollen joints at that visit). LDA, but not remission, was the key factor that determined whether to step-up to combination therapy at week 24. The SWEFOT study reported similar results with 29.8% of patients meeting EULAR criteria for response after 3 months of MTX monotherapy [44]. 

### 3.3. Ancillary Benefits

Besides its efficacy in ameliorating arthritis, MTX may have ancillary benefits. Patients with RA have a higher risk of cardiovascular disease, infection, and cancer [45,46,47], resulting in reduced survival. Low-dose MTX is reported to reduce all-cause mortality in RA by 60% and cardiovascular mortality by 70%, while other DMARDs have no effect on mortality. Protective effects appear to be independent of MTX dose [48]. Another study reported that all-cause mortality was reduced by 70% in a vast cohort of RA patients treated with MTX when compared to those receiving other treatments [49]. Protective effects were evident only after one year of MTX use and were independent of the effect on RA activity [49]. The biological mechanisms underpinning this protection are not fully understood. Effects of MTX on the survival of RA patients are likely to be mediated by its anti-inflammatory properties, since MTX failed to demonstrate any protective effects on cardiovascular events in patients with previous myocardial infarction or multivessel coronary disease who additionally had type 2 diabetes or metabolic syndrome but no major inflammation [50].

Interstitial lung disease (ILD) is a common extra-articular manifestation of RA [51], affecting around 10% of patients [52]. Chronic lung disease (CLD) and ILD increase the risk of death in RA patients [53,54]. Methotrexate has been associated with severe lung toxicity, including ILD [55]. However, there is recent data that questions the results of case studies or observational studies linking methotrexate to pulmonary damage. Two meta-analyses also failed to associate methotrexate with ILD; in the first one [56], a meta-analysis of clinical trials (22 studies with 8584 patients with RA), the use of MTX in patients with RA was associated with an increased risk of total respiratory complications (RR 1.10), mainly due to an increased risk of respiratory infections (RR 1.11) and acute pneumonitis (RR: 7.81) but it was not observed that treatment with MTX increased the risk of death due to lung disease (RR 1.53, 0.46–5.01). In the second one [57], a meta-analysis of the clinical trials of psoriasis, psoriatic arthritis, and inflammatory bowel disease, all clinical conditions without pulmonary manifestations, did not find an increase in lung disease in patients treated with MTX. A recent analysis of a large prospective cohort of predominantly male US Veterans showed no increase in mortality risk in RA-CLD patients treated with MTX or biologics [54]. Moreover, in a recent study, MTX was a strong predictor of survival in RA patients with ILD [53].

The role for MTX in the prevention of dementia remains controversial. Inflammation is a common feature of both RA and dementia [58]. Despite the blood brain barrier, systemic inflammation is associated with cerebral inflammation [58]. A retrospective population-based study involving more than 11,000 RA patients, including 70.6% of csDMARD users, revealed that csDMARDs users had a 40% risk reduction of dementia. The strongest effect was with the use of MTX [59]. 

Felty’s syndrome comprises a triad of RA, neutropenia, and splenomegaly, occurring in less than 1% of RA patients. Pseudo Felty’s syndrome is characterized by RA, monoclonal expansion of lymphocytes, and neutropenia. The main complication is infection, whose risk increases with the depth and duration of neutropenia [60]. MTX is the first-line treatment of Felty’s syndrome and is indicated, despite neutropenia [61,62]. 

### 3.4. Cost-Effectiveness

Therapeutic decisions should also be guided by cost-effectiveness considerations. Based on a decision analysis model extended to lifetime duration, early MTX monotherapy was more cost-effective than the early combination of MTX and TNF Inhibitor (TNFi)-based biologics [63]. The cost-effectiveness ratio of the early MTX strategy was less than $5,000/quality-adjusted life year (QALY) and more than $150,000/QALY for TNFis. In patients who failed to respond or to tolerate oral MTX; switching to SC MTX was described as more cost-effective than adding bDMARDs [64], since a large proportion of these patients achieved adequate response with SC MTX and, therefore, did not require more expensive therapy [65]. A cost-minimization analysis based on UK costs indicated that a routine use of SC MTX following oral MTX failure had the potential to save an estimated £7,197 (5536 US$) per patient in the first year of therapy [64]. Prefilled syringes and auto-injectors facilitate self-administration of SC MTX by the patients, thus increasing costs savings. In addition, in some countries, the prescription of bDMARDs can be reimbursed only if the patient did not adequately respond to at least 2 csDMARDs, if not contra-indicated [66,67].

### 3.5. Combinable with Other Treatments

MTX is a great combination partner with other csDMARDs and bDMARDs. The most popular csDMARDs combinations are MTX and HCQ and the triple combination of MTX, HCQ, and SSZ. The combination of MTX and HCQ is synergistic and gave better results than MTX alone in a head-to-head comparison after 6 months of treatment, but the difference was no longer significant after 12 months [68]. Combining MTX and HCQ may be useful in patients who have a good response to MTX, attaining LDA but not remission [69]. The efficacy of this combination may be explained by pharmacokinetic interactions. Concomitant administration of HCQ increases the mean area under the curve (AUC) of MTX blood concentration, decreases the maximum MTX concentration (Cmax), and extends the time to reach Cmax [67]. A reduced Cmax may also explain the diminution of acute liver adverse effects. However, extra vigilance for MTX adverse events is recommended, especially in patients with impaired renal function [67]. Triple therapy combining MTX, HCQ, and SSZ may be a valuable alternative to the addition of bDMARDs to MTX, in cases of inadequate response to MTX and in the absence of poor prognosis factors. In a randomized trial comparing a triple combination of csDMARDs versus a regimen of MTX and etanercept (ETN), patients receiving triple therapy adhered for a longer time to their regimen than patients receiving MTX + ETN, despite similar outcomes [70], thus reflecting better acceptability. Infections were more frequent with the MTX+ETN regimen, whereas gastrointestinal side effects were more frequent with the triple combination [71]. Conversely, in a network meta-analysis of 33 studies, the odds of remission were lower with triple therapy than with the combination of MTX and TNFi [72]. However, the analysis considered both patients with inadequate response to MTX and MTX-naïve patients. These results were contested by a recent meta-analysis showing that triple association of csDMARDs and the combination of MTX and bDMARD as a second-line treatment in patients with insufficient response to MTX gave similar results [69]. Nonetheless, this result provides additional evidence supporting conventional combinations over bDMARDs after an inadequate response to MTX. 

MTX is also a valuable partner in combined therapy with tsDMARD and bDMARDs. Such combinations have been shown to significantly improve clinical manifestations of RA [73,74,75,76]. Around two thirds of patients will have to step-up from MTX monotherapy to combined strategies [41]. In addition, RA patients with severe presentation and poor prognostic factors may require combined therapy upfront to prevent irreversible joint damage and functional impairment. The efficacy of targeted therapies is enhanced when the bDMARD is administered in combination with MTX. This may be due to complementary mechanisms of action, pharmacokinetic interactions, and reduction of the immunogenicity of the biologic agent.

The CONCERTO study was the first blinded, controlled trial to address the relationship between MTX dose and serum drug concentrations in RA patients treated with adalimumab. Steady-state trough serum concentrations of adalimumab were almost two-fold higher in patients concomitantly receiving MTX [77]. Patients were randomized into four groups with an increasing MTX dose (from 2.5/week to 20 mg/week, orally). There was no difference between the two highest MTX doses on clinical, radiographic, and functional response, whereas both doses were associated with better outcomes when comparing to the two lowest doses [77]. In parallel, adalimumab serum concentrations were higher with 10 or 20 mg MTX than with 2.5 or 5 mg MTX, mimicking the clinical findings. These results suggested that MTX at a dose of 10 mg/w reduced the clearance of adalimumab and increased the adalimumab serum level, thereby enhancing its efficacy. The MUSICA study [78] evaluated the effects of low and high MTX doses in combination with the initiation of adalimumab in patients who did not adequately respond to MTX. Patients with a stable MTX dose of at least 15 mg/week prior to screening were randomized to receive blinded MTX, at either 7.5 or 20 mg/week in combination with open-label 40 mg of adalimumab every other week. Since low-doses of MTX associated to adalimumab failed to show non-inferiority as compared to high doses for most clinical, functional, and ultrasound outcomes, the MUSICA study results do not support routine MTX reduction at the moment of adalimumab initiation. However, in a proportion of patients who may need to reduce MTX for toxicity reasons, much of the clinical efficacy in association with adalimumab may be retained, particularly when MTX dose reductions are modest.

Secondary failure to bDMARDs after an initial response can occur in as many as 30% of patients [79]. Development of anti-drug antibodies (ADAb) is one of the main drivers of loss of treatment efficacy [80,81,82,83,84]. ADAb have been described with infliximab, adalimumab, but not with etanercept. In a recent cross-sectional study on Spanish RA patients experiencing secondary failure while receiving TNFi, the prevalence of adalimumab ADAb was 29.3%, the one of infliximab ADA was 27.3%, and none of the patients treated with ETN developed ADAb [84]. Randomized-controlled trials reported ADAb in a small proportion on patients on golimumab and certolizumab [80]. ADAb either neutralize the active drug or decrease the serum drug concentration, resulting in a loss of clinical response [85]. Around 80% of patients who tested positive to ADAb had no detectable drug in the serum [84]. In a retrospective cohort of RA patients who were biologic-naïve at the time of enrolment, and were then treated with infliximab for a median time of 5.75 years, ADAb against infliximab were found in 33% of patients, in 24% of responders, and in all non-responder patients [83]. 

MTX may prevent [84,86] or reverse [87] the formation of ADAb and so maintain efficacy to TNFi, prolong drug survival, and prevent immune complex-mediated adverse events [80,83]. The pooled risk ratio for the formation of ADAb in patients receiving combined therapy with immunomodulators versus that of patients receiving anti-TNF monotherapy was 0.49 in a meta-analysis of 35 studies involving 6790 patients with inflammatory bowel disease [88]. The development of ADAb is reduced by the concomitant use of MTX in a dose-dependent manner [89]. It is, therefore, desirable to prescribe bDMARDs in combination with concomitant MTX when it is satisfactorily tolerated with toxicity. However, in the event of tolerability issues with MTX, the dose can be lowered while still providing efficacy benefits in combination, as illustrated in the CONCERTO [77] and MUSICA [90] studies.

### 3.6. Challenges Associated with MTX Use

When prescribing MTX as first-line therapy in RA patients, the physician has to face several challenges, such as explaining the time-course of the clinical benefits to the patient, improving patient compliance and adherence to therapy and monitoring, and avoiding unnecessary premature discontinuation of MTX are at the forefront of physician concerns.

The collective findings of combination therapy studies with MTX monotherapy arms, such as SWEFOT [44], CAMERA [14], TEAR [41], OPTIMA [91], and PREMIER [74], illustrate that, in general, it takes 6 months to see a full response to MTX. In patients with early RA and no poor prognostic factors, the relatively slow onset of action of MTX does not jeopardize clinical or functional outcomes. During this period, and especially during the first months, patients do not experience the full clinical benefit of MTX but are maybe exposed to adverse events. This is a crucial period in which too many patients discontinue MTX. The risk of premature stopping can be mitigated by the provision of clear and complete information to the patient. The slow kinetic of onset of symptomatic improvement with MTX can be mitigated by co-prescription of corticosteroids as a bridging therapy.

The best outcomes with the use of MTX are achieved when a shared decision-making approach to treatment is used in which the patient is an active participant. Patients need to understand and take responsibility for attendance for blood tests to ensure close monitoring of laboratory parameters, as well as following recommendations for folic acid supplementation in order to reduce the likelihood of certain adverse events. Reassuring tolerability and safety of low-dose MTX have recently been studied confirmed in the large placebo-controlled CIRT study aimed at determining if low-dose MTX could reduce the incidence of atherosclerotic events in patients with metabolic disorders and a history of myocardial infarction or multivessel coronary disease [50]. The study included 2391 patients who were allocated to the MTX arm. The median follow-up was 2.3 years before the study was stopped because of its futility. Most frequent AEs were infections, mouth sores and oral pain, unintended weight loss, leukopenia, and elevation of liver enzymes more than three times the normal range. Severe AEs are far less frequent. No case of marrow depletion was reported, even if leukopenia was frequent [50]. However, the report of more non-basal skin cancers in the MTX group was unexpected and deserves further exploration.

Elevations of liver enzymes must alert the physician to potential hepatotoxicity, in which case it may be prudent to temporarily reduce the MTX dose or even to discontinue the drug. Acute elevations of liver enzymes occur relatively frequently but are usually transient and generally resolve spontaneously. Rarely, persistent abnormal liver function tests reveal hepatotoxic effects of MTX, justifying treatment cessation. Among 41 patients receiving low-dose MTX for rheumatic diseases, experiencing elevation of liver enzymes, and underwent liver biopsy, only 2 patients had histological signs of direct hepatic MTX toxicity. Most frequently, liver biopsies revealed autoimmune hepatitis-like lesions, especially in patients with RA [92]. However, it is important to avoid the unnecessarily premature discontinuation of MTX for reasons of small elevations in transaminases. It is important to bear in mind other causes of transaminitis, such as alcohol consumption or over-the-counter use of nonsteroidal anti-inflammatory drugs (NSAIDs). The limited data available on the effects of alcohol consumption on the risk of liver toxicity in patients with RA who are receiving MTX are insufficient to draw firm conclusions on the amount of alcohol that patients receiving MTX can safely consume. International guidelines vary. The American College of Rheumatology recommends that alcohol should be avoided whilst on MTX [93], while EULAR advises avoiding MTX in patients with a history of alcohol abuse, without specifying a restriction of alcohol consumption otherwise [94]. A systematic literature review found little evidence to provide specific guidelines regarding alcohol consumption [95]. Following their analysis, they estimated a 3% risk for the development of severe liver disease in patients who drink over 100 g of alcohol (12.5 units) per week in the absence of additional risk factors such as obesity, diabetes, or hepatitis. The authors advised caution in using MTX in patients with risk factors. A more recent study suggested that a weekly consumption of <14 units of alcohol per week does not appear to be associated with an increased risk of hepatotoxicity as defined by transaminitis based on aspartate transaminase (AST) or alanine transaminase (ALT), >3 times the upper limit of normal. Consuming between 15 and 21 units is associated with a “possible risk”, and over 21 units with a significantly increased risk of transaminitis [96]. However, this definition of hepatotoxicity is somewhat arbitrary as it is not supported by the use of hepatic scans or histology. Furthermore, allowing alcohol consumption of up to 14 units/week may place patients at risk of hepatotoxicity, such as hepatic fibrosis and cirrhosis [97]. In practice, many rheumatologists allow patients without additional risk factors for hepatotoxicity to consume small to moderate amounts of alcohol while taking MTX, preferably avoiding alcohol consumption on the days that they take MTX. Decisions regarding alcohol consumption should be made on an individual patient basis after taking into consideration the presence of other risk factors. 

The next section of this position-paper will detail how to deal with these challenges in real-life conditions in order to get the most out of MTX as a first-line treatment.

## 4. How to Get the Most out of MTX as a First-Line Treatment?

Despite the many advantages of MTX, it may still be used suboptimally. Studies indicate that half of patients discontinue oral MTX within 2 years and that MTX is still frequently stopped rather than combined with bDMARDs or tsDMARDs when these target therapies are initiated. The risk of discontinuation is higher in older patients [98,99,100]. Patients should be treated early since a longer duration of RA decreases the probability of response [101]. Baseline high disease activity increases the probability of non-response in most studies [102,103].

When initiating MTX, the choice of dose, route of administration, and approach to subsequent dose escalation is not an exact science. Choices may depend on several factors, including the age, gender, ethnicity, weight, and renal function of the patient. These choices will be made with the intent to minimize any tolerability issues and drug-related toxicity, as well as to optimize efficacy and maximize adherence to MTX.

### 4.1. Dealing with Modifiable Predictors of Response to MTX

Some factors have been shown to impact the response to MTX. Of these, many such as age [104], gender [104], and pharmacogenetic profile [34] cannot be modified. However, others such as smoking may be modifiable, and physicians should endeavor to address this. 

Smoking may affect the pharmacokinetic and pharmacodynamics properties of MTX [25]. Smoking habits are predictors of poor response [102,104,105] in a dose-dependent manner [103], with heavy smokers being at higher risk of non-response. In addition, given the higher cardiovascular risk attached to RA patients, smoking cessation is highly desirable [106,107]. Even if it is a demanding objective [107], smoking cessation should be a priority for physicians.

Alcohol consumption, particularly when excessive, has been implicated in the risk of inadequate response to MTX [103]. For most patients, a modest alcohol intake is acceptable, provided that hepatic monitoring remains within safe limits.

Setting realistic but positive expectations about the likely magnitude of benefit, speed of onset, and mitigation strategies to limit toxicity and tolerability issues for MTX may favorably influence the drug’s efficacy via improved compliance. High quality education plays a crucial role in achieving this goal. The impact of patient anxiety has also been underscored in limiting the potential benefits of MTX and can be accentuated by the ready availability of misinformation through the internet and other sources. In a multivariate model predicting non-response to MTX in a real-life setting, the risk of non-response was higher in patients who were negative for rheumatoid factor, had a higher Health Assessment Questionnaire (HAQ) score, a higher tender joint count, a lower DAS28 score, and a higher anxiety score [108]. Nonetheless, anxiety is a modifiable risk factor. This issue should be addressed during the shared decision-making process. This process should include exploring the patient’s expectations regarding the disease and its treatment, providing honest and positive messages about achievable outcomes, and allaying unfounded fears about adverse effects of therapy. Educating the patient may lower the level of anxiety and improve clinical outcomes. 

### 4.2. Choosing the Route of Administration

When starting MTX, the physician has the choice between oral or parenteral administration. Although MTX can be given by intramuscular (IM) injection, the subcutaneous route is the preferred parenteral administration mode. 

Depending on the MTX dose, the bioavailability differs between oral and subcutaneous routes. Oral MTX at a dose of 7.5 mg/week has a 100 percent bioavailability. However, bioavailability is reduced by 30% starting at just 15 mg/week due to gut absorption limitations [22,109]. This threshold is also the point where the bioavailability of oral MTX reaches a plateau in RA patients [21,110]. Consequently, for a patient receiving a weekly dose of 20 mg, intestinal absorption may be reduced by 30% and the effective dose actually received by the patients may be only 14 mg/week. Despite this fact, orally administered doses greater than 15 mg/week are frequently used to control disease activity. 

By contrast, the bioavailability of SC MTX is greater than that of oral MTX and does not depend on the MTX dose with a linear increase in systemic exposure for increasing doses [21]. The ratio of dose-normalised AUC_0–24 h_ of the SC MTX compared to oral MTX was 127.61 (90% CI 122.30 to 133.15) in a cross-over study comparing the two administration routes of the same MTX dose [21]. The AUC ratio between SC and oral MTX increases with higher doses [110]. Therefore, upon switching from an oral to SC MTX formulation, a patient may experience a bioequivalent increase of more than 6 mg MTX per week. The practical implication of this is that for many patients, there may be no advantage to increasing oral MTX dose above 15 mg/week [21]. Rather, RA patients with an inadequate clinical response to orally administered MTX may benefit from the higher drug exposure offered when switched to the SC formulation [21]. Initiating the MTX regimen with SC administration has been associated with better clinical outcomes at the same dosages [111]. Initial treatment with SC MTX has been associated with lower rates of treatment change, no difference in toxicity, and some improvements in disease control compared to oral MTX over the first year, in a large cohort of early RA [112].

### 4.3. Starting at the Right Dose

As monotherapy, the dose of MTX can range from 7.5 to 25 mg/week, depending on national guidelines and physician’s preference. Such a range allows a wide variety of practices. Indeed, considerable heterogeneity exists in rheumatologist prescribing behaviours [113]. 

The recommended MTX dose at initiation is generally 10–15 mg/week [94] but should be personalized, depending on age, ethnicity, body weight and prior history of intolerance to other medications. In the interventional C-OPERA study involving Japanese patients, the initial MTX dose was 8 mg/week [114]. Considering the average patient body weight in the Japanese population, a MTX dose of 8 mg/week gave a similar MTX dose, per pound of body weight, to a 10 mg MTX dose in the USA or Europe [114]. In the French ESPOIR cohort involving 813 patients recruited in 14 regional centres the median MTX dose at initiation was 12.5 mg/week [113] with two peaks in the distribution at 10 mg and 15 mg/week. An optimal treatment defined by a starting dose of at least 10 mg/week during the first 3 months, with escalation to at least 20 mg/week at 6 months, has been associated with better clinical outcomes at two years in this cohort [113]. Of note, only 26.3% of patients were considered to have had an optimal dose. 

Clinical response is associated with longer-chain polyglutamates, which take 3–8 weeks to become detectable in erythrocytes [23]. These data raise the question as to whether a more rapid attainment of a steady state could be achieved with alternative dosing strategies, such as more rapid dose escalation or starting therapy with higher doses. Starting with a high oral dose does not make pharmacologic sense due to the reduced bioavailability of oral MTX above 15 mg/week. Recent trials have used higher initial MTX dosage (20–30 mg/week) [115]. However, combining the results of 31 studies involving 5589 patients, a meta-regression analysis did not support higher effectiveness of increasing MTX dose in monotherapy [116]. In the CONCERTO study which investigated the effects of starting with various dosages of oral MTX in combination with adalimumab 40 mg every other week, there was no difference in clinical or radiographic outcomes of patients starting with a 10 mg/week MTX dose, compared to those starting with a 20 mg/week dose [77]. Starting with higher oral doses may also result in discontinuation due to adverse events, such as nausea. 

In patients who reintroduce MTX after previous discontinuation for tolerability problems, the MTX dose selection should be determined by the last tolerated dose.

### 4.4. Escalating and Adapting the MTX Dose

In the context of a treat-to-target strategy, the large dose-titratable range of MTX offers numerous possibilities of dose adjustment before combining with bDMARDS and tsDMARDs. Around one third of DMARD-naïve patients will respond to MTX monotherapy and maintain favourable outcomes for several years as reported in the SWEFOT study [117]. The MTX dose should be increased until remission or at least LDA. In clinical trials comparing bDMARDs to MTX monotherapy, 25% of the patients allocated to the MTX arm reached ACR70 criterion within 6 months, bringing them in the range of LDA [118]. With the subcutaneous route [111], the proportion of patients reaching LDA was even higher. 

Titration is highly influenced by the starting dose and initial response to treatment. An optimal treatment defined by a starting dose of at least 10 mg/week during the first 3 months with escalation to at least 20 mg/week at 6 months has been associated with better clinical outcomes at two years in the ESPOIR cohort [113]. In the C-EARLY study comparing certolizumab + MTX and placebo + MTX in DMARD-RA patients with poor prognostic factors, MTX was started at 10 mg/week and escalated by 5 mg every 2 weeks, if tolerated, to a maximum of 25 mg/week by week 8. In the MTX monotherapy group, 39.4% of patients reached LDA at week 52. Withdrawals due to AEs occurred for 9.2% of patients [119]. These results suggest that the MTX dose should not be less than 10 mg/week and that dose escalation should be as rapid as tolerated. Maximum dose varies according to the region. The usual maximum MTX dose is 25 mg/week in USA and Europe although some patients may benefit from higher doses. The maximum recommended dose in China is 20 mg/week [120] and in Japan, 16 mg/week [114]. In a randomized controlled trial in patients with active RA despite oral MTX, intramuscular (IM) MTX, starting at a dose of 15 mg/week then increasing to 45 mg/week, did not improve disease control; however, higher doses were generally well tolerated [121]. The CRA 2012 Working Group recommends individualized dosing (oral or parenteral) titrated, to a usual maximum dose of 25 mg/week, by rapid escalation [6]. The French Society for Rheumatology recommends rapid dose escalation (e.g., 5 mg increments every 1–4 weeks) reaching an optimal dose of MTX (15 to 25 mg/week) within 4–8 weeks [122], depending on effectiveness and safety, as well as individual characteristics. The use of split oral dosing (2 half-doses at 8 h interval on one day of the week) has been reported to improve bioavailability, tolerance, adherence, and efficacy [123]. Given the higher bioavailability of SC MTX beyond 15 mg/week, as compared to oral MTX, switching to SC MTX before increasing the MTX dose beyond 15 mg/week could be good practice. In addition, switching to SC MTX may also prevent dose-dependent gastrointestinal side effects [124].

When remission without structural progression is achieved in a patient who is not taking glucocorticoid therapy, and sustained for many months or even years, therapy de-escalation according to tight control principles could be considered [122]. However, withdrawing MTX bears the risk of losing the state of remission and difficulties in regaining a good outcome after a post-withdrawal flare [118].

### 4.5. Switching to SC Route

In patients with an inadequate response or intolerance to oral MTX, parenteral administration should be considered [6]. In Germany, the 2012 adapted EULAR Task Force recommendations and treatment algorithm in RA support optimization of MTX monotherapy with parenteral administration [125]. Similarly, the British Society for Rheumatology 2008 guidelines for DMARD use recommends switching to IM or SC MTX if oral MTX is ineffective or not tolerated [126].

Parenteral administration has been reported to reduce disease activity in patients who have an inadequate response to oral MTX [127]. Conversely, a change from parenteral to oral MTX has been associated with disease flare [128], increased disease activity, and greater frequency of gastrointestinal side effects. Switching back to intramuscular MTX improved disease manifestations and reduced side effects [129]. 

Switching patients treated with oral MTX to an SC formulation should be considered when the maximum tolerated dose has been reached without achieving LDA. The SC route may also be proposed upfront to the patient at the time of MTX initiation, although this has relatively greater economic implications. However, data from a randomized controlled trial of 375 MTX-naïve RA patients favoured initiation of 15 mg once weekly MTX therapy by the SC route, which resulted in higher ACR20 and 70 response rates than oral MTX with a similar safety profile at 24 weeks [111]. Furthermore, in a large retrospective cohort study, Harris et al. reported that patients taking SC MTX received higher starting and maximum doses than those on oral MTX (>15 mg starting dose and >20 mg maximum dose) [130], perhaps reflecting the better tolerability of the parenterally delivered drug.

There have been advocates in Canada, Germany, and the United Kingdom for the use of SC MTX in order to optimize achievable efficacy of MTX prior to commencing biologic treatment or even to prevent or delay the need for biologic therapy with attendant health economic benefits [6,125,126]. Indeed, multiple lines of evidence suggest that SC MTX, which bypasses first-pass metabolism in the gastrointestinal tract, appears to be an effective option in patients who have had an inadequate response or are intolerant to oral MTX. Switching such patients to SC MTX has been shown to result in higher and more constant bioavailability [22], to be more effective at the same dosage [111], and to have less intense gastrointestinal side effects compared to oral MTX [124]. Higher bioavailability and increased long-chain MTXGlu_n_ may translate into efficacy [30,65,131,132]. A retrospective population-based study in 156 patients who were intolerant or unresponsive to oral MTX found that switching to SC MTX (*n* = 78) resulted in a decrease in RA disease severity, with good tolerability reported in these patients [65]. In the retrospective analysis of the US Veterans Affairs database of patients treated with injectable MTX after failing prior oral MTX, higher doses of MTX (>20 mg/wk) were achieved more readily with SC administration, and were associated with a significantly longer duration of MTX monotherapy before therapeutic change or the addition of other DMARDs/biologic agents, as compared to the oral formulation [133]. In the previously mentioned clinical trial comparing SC and oral MTX in 375 RA patients, after 16 weeks, patients from the oral group not fulfilling ACR20 criteria were switched from 15 mg of oral MTX to 15 mg of SC MTX following which 30% went on to achieve ACR20 responses [111]. In keeping with this observations, but in a real-world setting, retrospective analysis of 103 RA patients who switched from oral to SC MTX showed significant improvements in DAS-28 scores in patients who switched due to inefficacy or intolerability to prior oral MTX [132]. 

A prospective survey (i.e., office questionnaires) study evaluating patients (*n* = 70; each serving as their own control) with long-lasting RA who were switched from oral MTX to SC MTX due to side effects reported that when receiving the SC formulation, patients experienced less intense GI side effects, with no patients reporting vomiting or diarrhoea AEs [124]. 

SC MTX should also be considered in patients with poor compliance to oral MTX. There is currently no clear biomarker available to measure adherence to MTX in daily practice, despite some data suggesting a strong correlation between MTXGlu_n_ and compliance assessed by the electronic monitoring of treatment intakes with the Medication Event Monitoring System [134].

### 4.6. Giving Time for MTX to Achieve its Maximum Clinical Benefit and Using Bridge Therapies

Whether MTX is initiated at a higher dose, or a lower dose with upward titration, it will still take up to six months to achieve a maximum clinical benefit. Nevertheless, therapeutic response to MTX should be assessed after 3 months of treatment with the objective of a clinical improvement at least 50% [4]. If this short-term target is not reached, treatment adjustment should be considered.

Intra-articular glucocorticoids or short term systemic glucocorticoids may be used as part of the initial treatment strategy while waiting for MTX to take effect [6]. When MTX and intra-articular glucocorticoids were used with a treat-to-target approach in patients with early RA in the context of a clinical trial, this strategy effectively decreased synovitis, osteitis, and tenosynovitis and halted structural damage progression, as judged by MRI [135]. The EULAR Task Force recommends using glucocorticoids in combination with csDMARDs primarily as bridging therapy until the csDMARD reaches its maximum effect but corticosteroids should be tapered as rapidly as clinically feasible [4,6]. Intra-articular glucocorticoid application may be considered in residually inflamed or reactivated joint [4].

### 4.7. Preventing or Dealing with Adverse Events by Folic Acid Supplementation

In clinical practice, MTX-related toxicity may limit optimum treatment. Mild toxicity occurs in about 60% of patients and roughly seven to 38% of patients discontinue MTX within the first year of treatment due to toxicity [18,136,137]. Predisposing factors include existing folate deficiency, advanced age, cumulative MTX dose, renal insufficiency, and concomitant use of other folate inhibitors. A folate deficiency may cause side effects, such as mouth sores, abdominal pain, elevation of liver enzymes, or bone marrow depletion. Folate deficiency is frequent in RA patients and even more in those treated with MTX [138]. In a meta-analysis of six clinical studies, RA patients treated with low-dose MTX and “dummy” folic or folinic acid frequently experienced side effects: nausea and vomiting were reported by 35% of patients, abnormal liver blood tests were reported by 21% of patients, 22% of patients experienced mouth sores [139]. By contrast, genuine folic acid and folinic acid supplementation greatly reduced discontinuation of MTX compared to placebo supplementation in a placebo-controlled double-blind clinical trial. This was mainly due to the MTX stopping rules based on elevations of liver enzymes [137]. By delaying or preventing a premature switch from MTX to a far more expensive bDMARD or tsDMARD, folate supplementation may contribute to health costs savings [140]. 

There is a large variability of folate supplementation practices amongst rheumatologists. In a study of 2467 incident users of MTX in the Veterans Health Administration database, 27% of patients were not prescribed folic acid within 30 days of MTX initiation. After 20 months, only 50% of patients continued to receive folic acid [141]. 

In a Cochrane review of patients on MTX therapy for RA, folic acid or folinic acid supplementation reduced the risk of gastrointestinal side effects (nausea, vomiting, and abdominal pain) by 26% (not statistically significant); abnormal serum transaminase elevations were decreased by 77%; the risk of premature discontinuations from MTX for any reason was reduced by 61%. A trend towards a reduction in stomatitis was demonstrated but did not reach statistical significance [139]. Folate supplementation did not negatively impact the efficacy of MTX. These results were confirmed by a recent meta-analysis of seven studies involving 709 patients [142]. A third meta-analysis of 68 studies (not limited to RA) of patients taking MTX revealed that MTX increased the risk of nausea and vomiting, elevated transaminase levels, mucosal ulcerations, leucopenia, thrombocytopenia and infectious events. The concomitant prescription of folic acid or folinic acid was associated with a significantly lower risk of any adverse events [143].

No evidence of a significant difference between folic or folinic acid has been reported, but given its low-cost, folic acid may be the most cost-effective therapy [139]. Prescription of at least 5 mg folic acid per week with MTX therapy is strongly recommended [94] to avoid or prevent side effects. Taking folic acid two days before MTX intake may improve its tolerability. Folic acid supplementation can be increased to 5 mg/day, other than the methotrexate dosing day, if needed. However, a pilot study recently compared two folate supplementation regimens with folic acid (5 mg/week and 0.8 mg/week) with similar results [144]. Folinic acid should be administered on a weekly basis the day after MTX. Although daily use of folic acid other than the MTX day does not appear to affect MTX efficacy, dosing of folinic acid close to MTX administration or dose of folinic acid over 7.5 mg/week may hinder MTX efficacy.

### 4.8. Potential Toxicities

Most frequent adverse events of MTX are gastrointestinal and there is a correlation between the MTX dose and the intensity of side effects [124]. Other possible side effects include anaemia, neutropenia, increased risk of bruising and dermatitis. Gastric side effects can be reduced both by switching patients to the SC route [124] and by folate supplementation [139]. Elevation of transaminases is frequent, but MTX treated patients are not at increased risk of symptomatic or severe liver related adverse events [55]. The incidence of clinically important cytopenia in patients treated with low dose MTX is estimated to be less than 1%. A complete blood count, liver and renal biochemistry, and a chest radiograph should be ordered prior to initiating MTX therapy [6]. Screening for hepatitis B and C should be considered and HIV testing is recommended in high-risk patients [6].

MTX is excreted via the kidneys and is contraindicated in patients with an estimated glomerular filtration rate of less than 30 mL/min. Patients with impaired renal function should be closely monitored particularly for hematologic toxicity.

Concomitant intake of NSAIDs may reduce renal function, thereby increasing MTX bioavailability. In a registry-based analysis, concomitant use of NSAIDs and MTX increased the risk of serious adverse events with a higher risk of renal failure and cytopenia [145]. However, it should be remembered that NSAIDs, per se, may increase liver enzymes. Therefore, if raised transaminases are observed in patients taking concomitant MTX and NSAIDs, it is worth considering a decrease (or cessation) in the dose of NSAIDs rather than diminishing the MTX dose. The physician should pay attention to self-medication with NSAIDs. MTX should not be used with trimethoprim-sulfamethoxazole when used in a twice-daily regimen for treatment of an infection since the combination may result in significant bone marrow toxicity. However, it can be used for patients taking prophylactic doses of trimethoprim-sulfamethoxazole (dosed three times per week) for prevention of *Pneumocysis Carinii*. The main drug interactions are summarized in Table 1.

MTX-induced pneumonitis is a potentially life-threatening adverse effect [146]. It is an idiosyncratic hypersensitivity reaction due to activated T-cell-mediated (CD4 and CD8) stimulation of type 2 alveolar cells to release cytokines which lead to the recruitment of inflammatory cells, resulting in alveolitis. Acute or subacute pneumonitis related to methotrexate usually occurs during the first year of treatment [55,147]. It appears rapidly with low grade fever or high fever, nonproductive cough, and dyspnea that usually progresses to respiratory failure. The presence of eosinophilia in peripheral blood but not in bronchoalveolar lavage is common (50%). A diffuse interstitial pattern or a mixed interstitial-alveolar pattern (nodular or patchy infiltrates) is observed in the chest radiograph and high-resolution CT usually reveals the pattern of a usual interstitial pneumonia or a nonspecific interstitial pneumonia. Risk factors for this complication are age (>60 years), previous use of other DMARDs, hypoalbuminemia, the presence of diabetes mellitus, and a history of pleural disease and/or prior ILD related to AR (OR: 7.1), the latter being a great confounding factor when establishing a causal relationship with the drug [148]. It could be difficult to distinguish MTX-induced pneumonitis and pneumonitis related to *Pneumocystis* [149]. In a systematic review, 15 of 3463 RA patients (0.43%) who were receiving MTX developed MTX-induced pneumonitis [150]. Even if rare, this complication needs to be known and should not be neglected. Its treatment includes withdrawal of MTX, supportive therapy, and adjunctive steroids. The outcome is good if the condition is recognized early, and if appropriate treatment is given [146].

Although the overall benefit:risk ratio for MTX is remarkably favourable, as with any drug, adverse events can occur. Based on known adverse events of MTX treatment, we propose that it is good practice to assess the patient for several potential risk factors (Table 2). Among these precautions are the assessment of pre-existing infections, immunosuppression, haematological, kidney or liver diseases, neoplasms, the cardiovascular risks, potential interactions with concomitant therapies. In parallel the psychological status of the patient should be judged or assessed including the coping strategies and the internal resilience.

### 4.9. Placing MTX as an Anchor Drug

MTX is considered the initial drug of choice in RA pharmacotherapy with subsequent use as an anchor drug when used in combination due to its efficacy (when used optimally), safety, large dose-titratable range, and cost-effectiveness [4,5,94]. 

If the patient is unable to achieve treatment targets on maximally tolerated doses of oral MTX, and after a switch to the SC route, other treatment strategies must be considered. These include adding other csDMARDs, and adding or switching to biologic therapies or tsDMARDs, depending on the absence or presence of poor prognostic factors [4]. In routine practice, several combinations can be considered with some variations in preference according to country. As an example, physicians may take advantage of the combination with probenecid [151], the synergistic action of MTX and HCQ [69], or the triple combination MTX, HCQ, and SSZ [69,70]. In UK or Canada, rheumatologists are required to use at least two csDMARDs before the application of bDMARDs is approved by the payers [4]. The EULAR Task Force strongly recommends that bDMARDs and tsDMARDs should primarily be added to csDMARDs, such as MTX, since all bDMARDs have better efficacy when combined with MTX than as monotherapy [4]. This may be due to complementary mechanisms of action, pharmacokinetic interactions, and reduction of immunogenicity of the administered biologic agent.

If MTX is well tolerated in combination with concomitant csDMARDs or targeted therapies, the dose does not need to be tapered while waiting to achieve the desired treatment target. In case of poor tolerability, MTX can be used at 7.5–10 mg/week to provide additional efficacy to TNF-inhibitors [77] and intolerance at these low doses leading to discontinuation is very rare. Stopping MTX should be limited to patients with contraindications or very poor tolerance to MTX.

Current application of these guidelines is far from optimal. Analysing a database of 35,640 US patients with RA starting oral MTX, Rohr et al. [152] reported that over the 20,041 patients who had to change the treatment over a 5 year follow-up, only 13% switched to SC MTX. In those who added or switched to a biologic, only 37% were on >15 mg/week of oral MTX when a biologic was started. The addition or switch to a biologic occurred in the first 3 months of MTX therapy in 41% of patients, and in the first 6 months in 51% of patients. As stated by the authors, this study revealed that the MTX was dramatically underutilized as an efficacious anchor drug in clinical practice

### 4.10. Conception and Pregnancy

This section applies to RA patients who are planning to become pregnant and/or breastfeeding, men planning to conceive and patients who have accidentally conceived while taking MTX.

MTX is pro-abortive and teratogenic but does not decrease fertility [153]. Therefore, MTX at any dose should be avoided during pregnancy [154]. The optimal delay between the last MTX dose and conception is still a matter of debate and may vary between countries. Guidelines from the British Society for Rheumatology (BSR) and the British Health Professionals Society in Rheumatology recommend to stop MTX three months in advance of conception [154]. On the other hand, MTX is recommended to be stopped between 48 h and 6 months before conception, in France and in Spain, respectively. Thus, to avoid medicolegal issues, we recommend following the legal label of your country. In the case of accidental pregnancy on low-dose MTX, the drug should be stopped immediately [154].

MTX cannot be recommended in breastfeeding because [154] MTX is excreted into breast milk and may be toxic for the neonate.

If a woman has received MTX within 3 months prior to conception, or in the case of accidental pregnancy on MTX, folate supplementation should be continued prior to and throughout pregnancy at a dose of 5 mg/day [154]. 

Data regarding preconception exposure to MTX in men are reassuring, and, indeed, recent BSR guidelines state there is no need for men wishing to father children to stop MTX [154]. A study involving a very large cohort concluded that paternal exposure to MTX within 90 days before pregnancy was not associated with congenital malformations, stillbirths, and preterm birth [155]. Older recommendations suggest to stop MTX three months prior to conception, but this is not evidenced by an understanding of the impact of MTX on spermatogenesis or paternal-mediated teratogenicity, but rather relies on the timeframe of spermatogenesis [156].

### 4.11. Vaccinations

RA patients are more prone to infections than healthy subjects due to the immune dysfunction associated with the condition. DMARDs inhibit cellular and humoral immunity, thus aggravating the susceptibility to infections. For these reasons, vaccines against preventable diseases should be counselled in patients with RA. Influenza, pneumococcal, and shingles are important for all patients with RA whereas human papilloma virus, hepatitis B virus, and yellow fever (YF) vaccines are relevant only in selected patients, such as those living in or traveling to a YF-endemic country [157]. Protection is far from optimal. In 2012, in the United States, only 28.5% of patients older than 60 with rheumatic diseases were vaccinated against pneumococcal pneumonia; 45.8% were optimally vaccinated against influenza, and only 4.0% of patients were vaccinated against shingles [158]. 

MTX significantly decreases vaccine response to pneumococcal [159,160] and seasonal influenza vaccines [159] in a dose-dependent manner [161]. A recent randomized controlled trial showed that, in order to impair the response to vaccines, MTX had to be present just before or at the same time of vaccination [162]. It is, therefore, recommended that vaccination be done before a DMARD is started, where practically feasible [5]. However, it may be necessary to prioritize control of the disease. In such circumstances, and for seasonal vaccinations, MTX dosing can be temporarily interrupted. In a prospective randomized parallel-group trial, patients with RA taking a stable dose of MTX were randomly assigned at a ratio of 1:1:1:1 to continue MTX (group 1), suspend MTX for 4 weeks before vaccination (group 2), suspend MTX for 2 weeks before and 2 weeks after vaccination (group 3), or suspend MTX for 4 weeks after vaccination (group 4). All participants were vaccinated with trivalent influenza vaccine. Temporary MTX discontinuation improved the immunogenicity of vaccination. No difference was found between groups three and four [162]. These results were confirmed in another study [161] that demonstrated improvement in immunogenicity of seasonal influenza vaccination in patients receiving methotrexate in whom the drug was withheld for 2 weeks immediately after immunisation, compared with those who continued to receive methotrexate with a satisfactory vaccine response seen in 76% of the former compared with 55% of the latter group. However, there was a slightly higher incidence of flares in those patients who withheld methotrexate compared with those who had remained on methotrexate (11% vs. 5%). Therefore, suspending MTX administration for 2 weeks after the vaccination seems to be good practice. 

Shingles vaccine can be used safely with low-dose MTX (i.e., up to 25 mg/week) [157]. 

Live vaccines, such as yellow fever, measles/mumps/rubella, varicella, and oral typhoid vaccinations, are contra-indicated in patients receiving MTX. Information remains too scarce to question the current guidelines.

### 4.12. No Need for MTX Discontinuation in Case of Surgery

Despite the efficacy of DMARDs, 58% of RA patients will ultimately undergo orthopaedic surgery, with nearly 24% undergoing large-joint arthroplasty [163]. Patients receiving DMARD are potentially at higher risk of infections and delay in wound healing due to the anti-inflammatory properties of these compounds. A study involving 388 patients with RA who underwent surgery found that patients who continued MTX through surgery had fewer complications, infections, and RA flares than patients who discontinued MTX for 2 weeks before and after surgery [164]. A special consideration should be given to patients who develop renal dysfunction postoperatively, since the risk of MTX toxicity is increased in cases of renal failure.

### 4.13. Sharing the Decision Making with the Patient

Adherence to treatment has been shown to depend on the level of appropriate information provided to patients and the amount of good interaction with the rheumatologist [165]. Non-adherent patients flare four times more frequently than adherent patients [100]. Most arthritis patients prefer to be involved in decisions about their medication [166], and this has been linked to higher patient satisfaction.

Patients exhibit a complex range of beliefs related to DMARD therapy [167]. Furthermore, the degree to which patients desire to be directed by their physician regarding treatment choice varies tremendously and may evolve over time to become more collaborative [168]. Sympathetic understanding will facilitate the provision of appropriate information to allow the patient to make informed decisions [169]. Furthermore, it is crucial to educate patients about the importance of a T2T strategy, since it has been shown that patient preference is among the leading barriers to treatment adjustment to T2T in RA [170].

The EULAR Task Force recently reiterated the relative safety of MTX and recommended that the frequent fears of patients after reading the package insert should be addressed by providing appropriate information [4]. The overemphasis on the risk of side effects that are widely spread on social media can generally be overcome by an open, balanced, and reassuring discussion regarding the benefits/risks and risk mitigation at the time of the first prescription. The other crucial points to address are the importance of maintained therapy, especially in those patients in remission who want to stop MTX, and patient adherence (in particular, for patients who tell their practitioner that they are taking MTX when, actually, this is not the case). Available data suggest that adherence to RA therapy does not exceed 66% [171], but this proportion is enhanced by the belief in the necessity of the medication. For this reason, when explaining the achievable goals of treatment to the patient, the rheumatologist should emphasise the availability of long-term observational data demonstrating the numerous benefits and good overall tolerability of MTX.

When initiating MTX, we recommend that rheumatologists adopt a shared decision-making approach to the recommended regime. Key points to be emphasised in dialogue with the patient are summarized on Table 3.

## 5. Conclusions

In conclusion, MTX is a remarkable drug with a unique place in the pharmacotherapeutic management of RA. Key recommendations for clinical use are listed in Table 4. In the last 2 decades, the cumulative experience of rheumatologists has helped to refine the possibility of achieving the best outcomes with MTX. It has a large dose titratable range and can be administered as a once weekly dose regimen by either oral or subcutaneous routes. Parenteral administration of MTX has the advantages of maximising bioavailability, reducing gastrointestinal intolerance, and potentially enhancing compliance and adherence. MTX is recommended as the first-line csDMARD treatment for RA and can also be used in combination with other csDMARDs, bDMARDs, or tsDMARDs. In the case of concomitant use with targeted therapies, MTX facilitates optimum achievable efficacy, and also has the advantage, when used with bDMARDs, of reducing ADAb responses to administered biologic. MTX is also highly cost-effective and has a favourable benefit/risk profile overall. Nonetheless, adverse events associated with MTX are well-described and tolerability issues can be a troublesome complication of this treatment. However, with appropriate risk-mitigation strategies, including folic acid supplementation, blood monitoring, and patient education, one can optimise achievable outcomes.

## Figures and Tables

**Table 1:** Drug interactions. MTX, methotrexate.

Interactions	Source of Interactions
Increase MTX levels	Allopurinol, triamterene Decrease renal MTX clearance: ciprofloxacin, cephalothin, penicillin, probenecid, sulfonamides Decrease MTX excretion: diuretics, proton pump inhibitors Increase MTX reabsorption by the kidney tubule: probenecid
Decrease MTX levels	Decrease intestinal absorption of MTX: chloramphenicol, tetracyclines
Increase the risk of bone marrow suppression	Chloramphenicol, co-trimoxazole, pyrimethamine, sulfonamides, trimethoprine-sulfamethoxazole
Increase liver toxicity	Alcohol, leflunomide

**Table 2:** Preparation of MTX therapy.

Diagnosis of rheumatoid/inflammatory arthritis and/or need for treatment
Diagnosis of chronic kidney or liver diseases
Assessment of cardiovascular risks
Assessment of a neoplastic disease
Discussion on smoking habits and the advantage of smoking cessation
Evaluation/judging of depression and resilience/coping strategies
Assessment of anemia, leukopenia, or thrombocytopenia
Documentation of concomitant drug therapy
Defining therapeutic aim
Shared decision making with the patient
Evaluation of alcohol-consumption
Diagnosis of active/chronic infection with herpes zoster, tuberculosis, hepatitis, HIV, relevant fungal infection
Diagnosis of immunodeficiency
Documentation of vaccination status
Consider testing for tuberculosis
Anticonception/assessing wish of conception/family planning

**Table 3:** Key points patients will need to be counselled about when rheumatologists adopt a shared decision-making approach to the recommended regime. NSAIDs, non-steroidal anti-inflammatory drugs.

Topics to Discuss with Patients
• The choice of routes of administration and the advantages of each
• Expected time to experiencing benefits (this is very important as a patient may experience transient tolerability problems such as nausea prior to experiencing and improvement in symptoms and there will therefore be a danger of non-adherence if there has not been appropriate counselling)
• Potential toxicities with reassurance about the capability to mitigate these risks through regular and appropriate blood monitoring
• The potential tolerability issues, especially nausea with appropriate reassurance that this can be lessened or mitigated with use of folic acid supplementation or parenteral administration
• Women of childbearing potential need to be counselled about pregnancy and family planning.
• Vaccinations and how to ensure optimum outcomes of vaccination
• Alcohol consumption
• Interactions between MTX and other medication with particular advice about NSAIDs

**Table 4:** Key recommendations for clinical use of MTX. csDMARD, conventional synthetic disease-modifying antirheumatic drugs; bDMARDs, biological DMARDs; tsDMARDs, targeted synthetic DMARDs; SC, subcutaneous.

Key Points for Clinical Use of MTX
**Modify predictors of response to MTX**
Encourage smoking cessation
Limit alcohol consumption
Ensure appropriate education on how to optimise outcomes
Manage anxiety and depression
**Start at the right dose**
Generally 10–15 mg/week, but should be personalized There is no need to start with higher dose
Choose between oral and parenteral route.
**Escalate as quickly as tolerated**
Titrate dose according to individual clinical response Generally aim to reach a dose of at least 20 mg/week after 6 months Consider local recommendations and clinical context
Switch to SC route for doses higher than 15 mg/week to enhance MTX bioavailability
**Prevent side effects**
Start folic acid supplementation at a dose of at least 5 mg/week and up to 5 mg/day other than the day of methotrexate (or folinic acid at a dose lower than 7.5 mg/week)
Instruct the patient to not take folic acid on the day of MTX administration
Folinic acid should be administered on a weekly basis the day after MTX administration
Counsel patients about risk mitigation through blood monitoring according to local protocols
**Be aware of potential drug to drug interactions**
Instruct the patients to seek advice from their rheumatologist about compatibility with methotrexate of self-medicated drugs or drugs prescribed by another physician
**In case of poor tolerability**
Diminish gastrointestinal side effects by switching to SC route
**In case of inadequate response**
Enhance MTX bioavailability by splitting oral dose or switching to SC route
Consider combination with csDMARD, bDMARDs or tsDMARDs Do not stop MTX nor reduce MTX dose unless there are toxicity or tolerability concerns
**Give time for MTX achieving maximum clinical benefit**
Use intra-articular or systemic glucocorticoids as bridging therapies
Educate the patient and adopt a shared decision-making approach
